# Normalization of Tumor Microenvironment by Neem Leaf Glycoprotein Potentiates Effector T Cell Functions and Therapeutically Intervenes in the Growth of Mouse Sarcoma

**DOI:** 10.1371/journal.pone.0066501

**Published:** 2013-06-13

**Authors:** Subhasis Barik, Saptak Banerjee, Atanu Mallick, Kuntal Kanti Goswami, Soumyabrata Roy, Anamika Bose, Rathindranath Baral

**Affiliations:** 1 Department of Immunoregulation and Immunodiagnostics, Chittaranjan National Cancer Institute (CNCI); Kolkata, India; 2 Department of Molecular Medicine, Bose Institute, C.I.T. Scheme, Kolkata, India; University of Pittsburgh, United States of America

## Abstract

We have observed restriction of the murine sarcoma growth by therapeutic intervention of neem leaf glycoprotein (NLGP). In order to evaluate the mechanism of tumor growth restriction, here, we have analyzed tumor microenvironment (TME) from sarcoma bearing mice with NLGP therapy (NLGP-TME, in comparison to PBS-TME). Analysis of cytokine milieu within TME revealed IL-10, TGFβ, IL-6 rich type 2 characters was switched to type 1 microenvironment with dominance of IFNγ secretion within NLGP-TME. Proportion of CD8^+^ T cells was increased within NLGP-TME and these T cells were protected from TME-induced anergy by NLGP, as indicated by higher expression of *p*NFAT and inhibit related downstream signaling. Moreover, low expression of FasR^+^ cells within CD8^+^ T cell population denotes prevention from activation induced cell death. Using CFSE as a probe, better migration of T cells was noted within TME from NLGP treated mice than PBS cohort. CD8^+^ T cells isolated from NLGP-TME exhibited greater cytotoxicity to sarcoma cells *in vitro* and these cells show higher expression of cytotoxicity related molecules, perforin and granzyme B. Adoptive transfer of NLGP-TME exposed T cells, but not PBS-TME exposed cells in mice, is able to significantly inhibit the growth of sarcoma *in vivo*. Such tumor growth inhibition by NLGP-TME exposed T cells was not observed when mice were depleted for CD8^+^ T cells. Accumulated evidences strongly suggest NLGP mediated normalization of TME allows T cells to perform optimally to inhibit the tumor growth.

## Introduction

It has been widely recognized, though recently, that a tumor grows toward a malignant phenotype by altering its microenvironment in a way that accelerate its malevolent potential [1]. Consequently, current anti-cancer therapeutic strategies not only target the intracellular distortions of a malignant cell but also cause deformation in its extracellular microenvironment [2]. The remedial strategies are designed with a major aim to optimize the antitumor immunity in the tumor vicinity, as the alterations in the tumor microenvironment (TME) are dominantly manifested as an impaired immune response. As tumor is seeded in the soil, i.e., TME, its progression, and, in some cases regression solely depends on the constituents of TME [3]. TME, in addition to tumor cells, comprised of immune cells, fibroblasts, endothelial cells, perivascular cells and the extracellular matrix [4]. These components interact with each other and contribute significantly in tumor progression. Cells (tumor associated macrophages (TAM), myeloid derived suppressor cells (MDSC), tumor associated dendritic cells, mesenchymal stromal cells and tumor cell itself) within TME produce several angiogenic growth factors, like, VEGF, PDGF, TGFβ and HGF that stimulate tumor cell proliferation [5], [6]. Contrariwise, as tissue becomes cancerous, pathological interactions between cancer cells and host immune cells in the TME create an immunosuppressive network that protects the tumor from immune attack [7] leading to tumor growth, progression, invasion and metastasis [8]. Thus, normalization of TME is a principle task of cancer immunotherapy.

The neem (*Azadirachta indica*) tree has been found as a traditional medicinal plant from ancient Harappa and Mohenjo-Daro civilizations in Indian subcontinent. Neem received the key interest in traditional medicine as SARBOROGANIBARANI (can cure all forms of diseases) [9], [10]. In 1992, US National Academy of Science designated this tree, as ‘A tree sloving global problem’ (National Research Council, 1992). Because of its tremendous therapeutic, domestic, agricultural and ethnomedicinal significance, and its proximity to human culture and civilization, neem has been called “the wonder tree” and “nature's drug store.” All parts of this tree, particularly the leaves, bark, seed-oil and their purified products are attempted to use for the management of different forms of cancer [11], however, in depth mechanistic study is lacking in most of the cases.

In prophylactic settings, we have reported that neem leaf preparation [12], [13] and its active principle neem leaf glycoprotein (NLGP) [14] (a nontoxic preparation from neem leaf [15]), can effectively prevent the growth of murine carcinoma and melanoma. NLGP activates T cells [16], [17], NK cells [18], inhibits suppressor Tregs [19], promote type 1 cytokines [20], [21] and maintains type 1, anti-tumor chemokine milieu [22], [14], thereby, induces antigen specific tumor killing, as documented *in vitro* [13] and *in vivo* [16], [23], [24]. We have recently reported that NLGP is also able to restrict the sarcoma growth in therapeutic settings, where NLGP activated CD8^+^ T cells play a pivotal role [25]. As CD8^+^ T cells are impregnated within TME, it might obtain some unexplored stimulatory or suppressive signals from their environment, crucial for tumor induced dysregulation of T cell functions. Accordingly, modulatory role of NLGP on sarcoma TME is objected to evaluate here to know their influence on CD8^+^ T cell functions, thereby, restriction of sarcoma growth.

## Results

### NLGP normalizes cytokine milieu within TME to type 1

**Figure 1:**
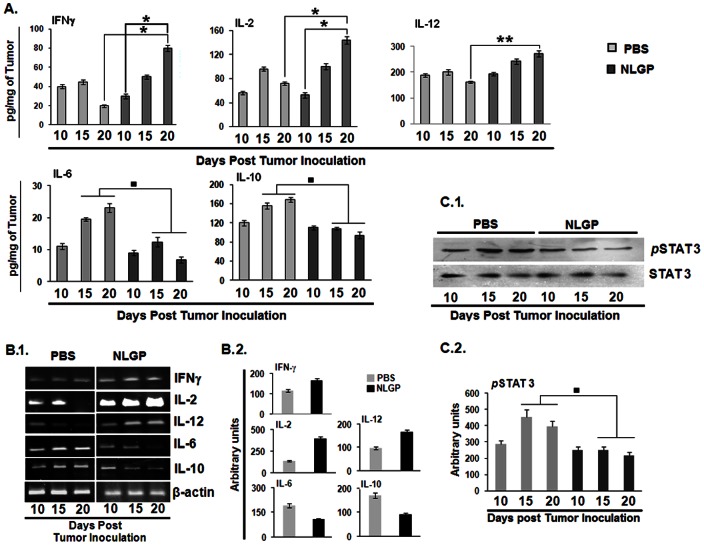
NLGP modulates immunosuppressive cytokine milieu within TME. A. Sarcoma 180 tumor tissues (100 mg) harvested from Swiss albino mice was lysed by freeze-thaw technique in 1 ml PBS supplemented with a cocktail of protease inhibitors. Tumor tissue lysates, representing TME from either PBS or NLGP treated mice (n = 6 in each case) were assessed for IFNγ, IL-2, IL-12, IL-6 and IL-10 by ELISA. Cytokines were quantitated as pg/mg of tumor tissue ± SE. *p<0.001, **p<0.05, in comparison to PBS treated tumor on day 15 and 20. **B.1.** Total RNA was isolated from tumor of PBS and NLGP treated mice (n = 6 in each case) to analyze genes of IFNγ, IL-2, IL-12, IL-6 and IL-10 by RT-PCR. **B.2.** Densitometric analysis was performed in each case, ▪*p*<0.01. **C.1.** STAT3 and *p*STAT3 levels were studied in total protein isolated from PBS and NLGP tumors (n = 3, in each case) by Western blot analysis, **C.2.** and data from three individual observations was analyzed by densitometric scanning, in comparison to PBS treated tumor on day 15 and 20, ▪*p*<0.01.

As TME is dominated with immunosuppressive cytokines, like, IL-10, TGFβ etc. with low IFNγ and IL-12, normalization of TME is desired to initiate antitumor immune response, especially T cell response. For this purpose, two groups of mice with established sarcoma (average tumor volume, 100 mm^3^) were treated with NLGP and PBS respectively. Tumors were harvested in three different days (n = 3, in each time point) following initiation of the NLGP treatment and cytokine secretory status were assessed from cellular contents by ELISA and immunoblotting. Tumors of NLGP treated mice exhibited significant upregulated level of IFNγ, IL-2 and IL-12 (Figure 1A, B.1 and B.2) in day dependent manner and reduced release of IL-6, IL-10 and TGFβ (Figure 1A, B.1 and B.2) within TME, in comparison to mice having PBS injection. In accordance with our earlier observation on NLGP mediated downregulation of phospho STAT3 [19], here, we also observed day dependent downregulation of the same within TME from NLGP treated mice (Figure 1C.1 and C.2).

### NLGP normalizes protumor angiogenic and hypoxic TME

**Figure 2:**
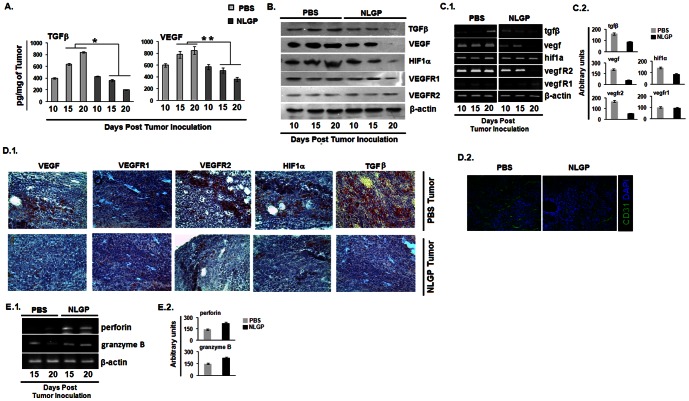
NLGP normalizes protumor angiogenic and hypoxic TME. Sarcoma 180 tumor tissue (100 mg) harvested from Swiss albino mice was lysed by freeze-thaw technique in 1 ml PBS supplemented with a cocktail of protease inhibitors. **A.** Tumor tissue lysates, representing TME from either PBS or NLGP treated mice (n = 6 in each case) were assessed for TGFβ and VEGF by ELISA. Cytokines were quantitated as pg/mg of tissue ± SE. *p<0.001, **p<0.01, in comparison to PBS treated tumor on day 15 and 20. **B.** Total protein was isolated from PBS and NLGP treated tumors (n = 3 in each case) to assess TGFβ, VEGF, HIF1α, VEGFR1, VEGFR2 and β-actin by Western blot analysis **C.1.** Total RNA was isolated from tumors of PBS and NLGP treated mice (n = 3 in each case) to analyze genes, like, VEGF, HIF1α, VEGFR1, VEGFR2 and TGFβ at transcriptional level by RT-PCR **C.2.** Densitometric analysis was performed in each case. Frozen sections of tumors from either PBS or NLGP treated mice were stained **D.1.** immunohistochemically with monoclonal antibodies, specific for VEGF, VEGFR1, VEGFR2, HIF1α and TGFβ and **D.2.** with fluorescence tagged anti-CD31 antibody **E.1.** Total RNA from tumors of PBS and NLGP treated mice was used to determine the status of perforin, granzyme B on day 15 and 20 of tumor inoculation. **E.2.** Densitometric analysis was performed in each case.

Type 2 cytokine bias, along with promoted angiogenesis within TME, is critically required for tumor promotion [26]. In order to understand the status of angiogenic TME, we initially examined the pro-angiogenic molecules (VEGF, TGFβ and HIF1α) within tumors from PBS and NLGP treated mice. Results of ELISA, Western Blot and RT-PCR analysis suggested that quantity of all of these molecules were significantly less in tumors from NLGP treated sarcoma bearing mice in day dependent manner (Figure 2A,B,C1 and C2). To confirm these results further, we performed immunohistochemical study on VEGF, TGFβ, VEGFR1, VEGFR2 and HIF1α to check their differential expression in tumors. Tumors from NLGP treated mice show reduced expression of VEGF, TGFβ, VEGFR2 and HIF1α, however, the level of VEGFR1 remains unchanged in NLGP treated group in comparison to controls (Figure 2D.1). As angiogenesis is directly correlated with the expression of CD31 on vascular endothelial cells, CD31 was assessed within tumor tissues by immunofluoroscence. NLGP treated group showed significantly reduced level of CD31^+^ cells than PBS controls (Figure 2D.2).

### NLGP normalizes suppressed cytotoxic gene expression of CD8^+^ T cells

Treatment with NLGP in therapeutic settings enhances the frequency of activated CD8^+^ T cells within TME and their cytotoxic efficacy [27]. Assessment on the status of genes associated with cytotoxic functions revealed significant upregulation in perforin and granzyme B in purified CD8^+^ T cells from cellular preparation of NLGP treated mice tumors (Figure 2E.1 and E.2).

### NLGP reduces suppressor cell frequency and tolerogenic molecules

**Figure 3:**
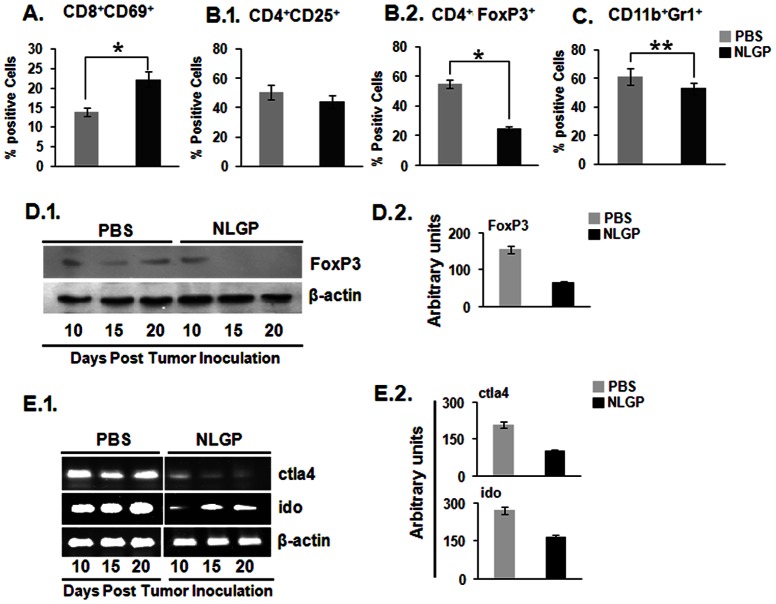
NLGP downregulates suppressor cells within TME. Single cell suspension was prepared from day 20 tumors of PBS and NLGP treated mice. **A.** Proportion of activated T cells **B.1.** CD4^+^CD25^+^ cells, **B.2.** Tregs, **C.** and MDSCs within tumors from these two groups of mice was determined flow cytometrically after labeling cells with fluorescence tagged CD8, CD69, CD4, CD25 and CD11b, GR1 antibodies. *p<0.001, **p<0.05. **D.1.** Total protein was isolated from tumors at different days and level of Foxp3 was analyzed by immunoblot analysis. **D.2.** Densitometric analysis was performed in each case. **E.1.** Total RNA was also purified to assess the expression status of CTLA4 and IDO on different time points of tumor growth. **E.2.** Densitometric analysis was performed in each case.

During tumorigenesis, enhanced frequency of suppressor cells, like, Tregs, MDSCs and TAMs, make the situation more favorable for tumorigenesis. These cells negatively interfere in optimum anti-tumor T cell functions. Here, we have evaluated the activation status of CD8^+^ cells, along with Tregs and MDSCs within TME of NLGP treated mice. In comparison to PBS treated controls, NLGP activates CD8^+^ T cells, as denoted by their higher expression of CD69 molecules on cell surfaces (Figure. 3A). Number and per cell expression of CD4^+^Foxp3^+^ Tregs (Figure 3B.1 and B.2) and CD11b^+^Gr1^+^ MDSCs (Figure 3C) within NLGP treated TME was reduced, but difference is not statistically significant. Western blot analysis also showed the downregulation of Foxp3 within NLGP-TME (Figure 3D.1 and D.2). In addition, it was observed that TME from NLGP treated mice showed lower mRNA expression of IDO1 and CTLA-4 (Figure 3E.1 and E.2) in comparison to PBS-TME.

### NLGP normalizes loco-regional chemokine network

**Figure 4:**
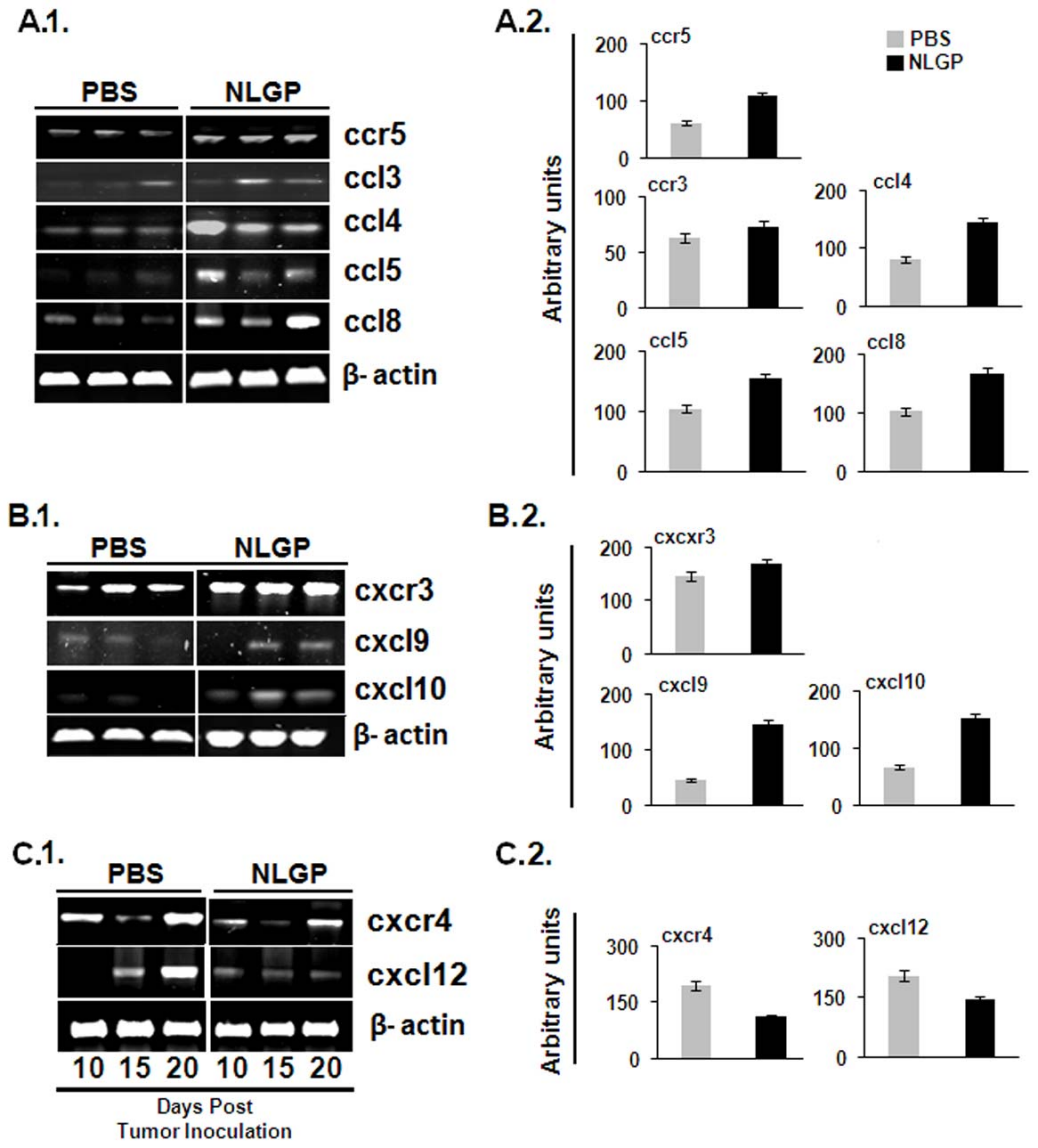
NLGP normalizes chemokine network within TME. Total RNA was isolated from tumors of PBS and NLGP treated mice. cDNA was prepared from RNA and PCR was performed for different chemokine related genes, **A.1.** ccr5, ccl3, ccl4, ccl5, ccl8; **B.1.** cxcr3, cxcl9, cxcl10; **C.1.** cxcr4, cxcl12. **A.2, B.2, C.2.** Densitometric analysis was performed in each case.

Chemokine-mediated T cell migration is essential for optimal anti-tumor immune response [28]. Similar to the cytokine network, chemokine milieu is also dysregulated within TME, thus, hampers movement of effector CD8^+^ T cells in tumors. In an objective to know the status of chemokine receptor-ligand profile, we have analyzed different genes related to chemokine ligands (ccl3, ccl4, ccl5, ccl8, cxcl9, cxcl10 and cxcl12) and chemokine receptors (ccr5, cxcr3 and cxcr4) at transcriptional level. In our findings, we observed chemokine receptor cxcr3 and ccr5 were upregulated in NLGP treated tumors (Figure 4A and B). At the same time it was found that ligands for ccr5 like ccl3, ccl4, ccl5 and ccl8 were upregulated within NLGP-TME (Figure. 4A.1 and A.2), although extent of upregulation is varied. Same trend of upregulation was observed in case of cxcr3 ligands, cxcl9 and cxcl10 (Figure 4B.1 and B.2). We also observed a marked downregulation of cxcr4 and cxcl12 in case of NLGP treated tumor than PBS controls (Figure 4C.1 and C.2).

### NLGP modulated TME enhances CD8^+^ T cell functions

It is evident from above mentioned results that NLGP is effective to normalize TME. Accordingly, the question is asked how CD8^+^ T cells work within this NLGP normalized TME. For this purpose, MNCs were purified from spleen of disease free mouse and exposed to PBS-TME and NLGP-TME for 120 hrs. CD8^+^ T cells were then purified from exposed MNCs and subjected to following functional assays.

### NLGP normalized TME activates and proliferates effector T cells

**Figure 5:**
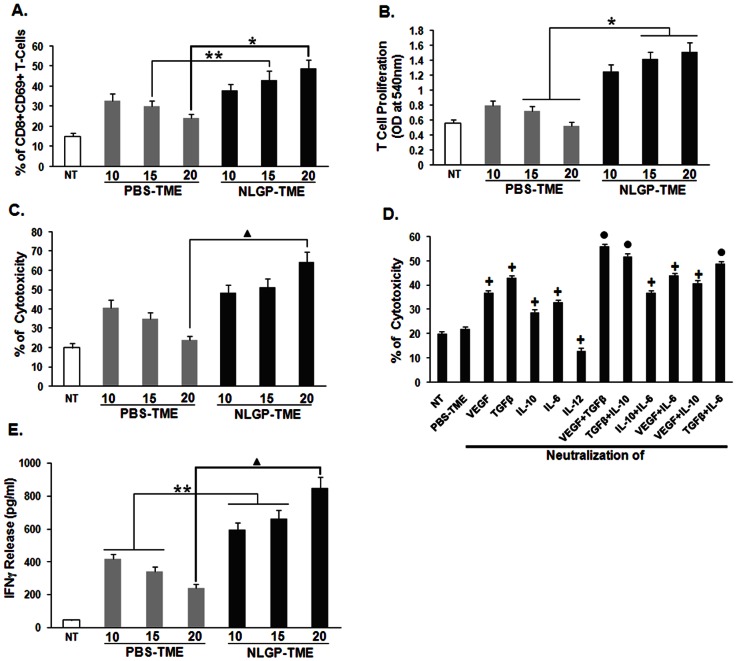
NLGP normalizes T cell functions within TME. Lysates were prepared from tumors of PBS and NLGP treated mice, designated as PBS-TME and NLGP-TME. MNCs from normal mice were incubated with PBS-TME and NLGP-TME, prepared from tumors of different days for 120 hrs. **A.** percentage of CD8^+^CD69^+^ T cells was analyzed within different TME treated MNCs * *p*<0.01, ** *p*<0.05. **B.** CD8^+^ T cells were then allowed to proliferate for 72 hrs and proliferation was determined by MTT assay. NLGP stimulation in MNCs was kept as control. *p<0.01. **C.** After 120 hrs of incubation with different TMEs, CD8^+^ T cells were purified by MACS. Cytotoxicity of these cells towards sarcoma 180 cells was assessed by LDH release assay. ^▴^ *p*<0.01. **D.** Different growth factors (VEGF, TGFβ) and cytokines (IL-10, IL-6 and IL-12) in single or in combination were neutralized within PBS-TME (Tumor lysate prepared from tumor of day 20) using their respective antibodies. Splenic MNCs were then exposed to differentially neutralized PBS-TME and after 120 hrs incubation CD8^+^ T cells were purified by MACS. Cytotoxicity of these differentially exposed CD8^+^ T cells towards Sarcoma 180 cells was measured. ^•^ *p<*0.001, **^+^** *p*<0.01 in comparison to PBS-TME. **E.** CD8^+^ T cells purified in similar fashion as described in C and cultured for 48 hrs. Cell supernatants were used to measure IFNγ level by ELISA. NLGP stimulation in normal CD8^+^ T cells was kept as control. *p* = 0.0023. In every case, comparison was made between PBS-TME exposed T cells vs same exposed to NLGP-TME on day 15 and 20, ^▴^p<0.01, **p<0.05.

Activation and proliferation of CD8^+^ T cells are essential to impart anti-tumor immunity [29]. To validate this hypothesis; we have checked the expression of activation marker, CD69 on CD8^+^ T cells. It was observed from experimental results that due to exposure of T cells to NLGP-TME, higher proportion of CD69 marker was expressed on T cells, in comparison to those cells exposed to PBS-TME (Figure 5A). These cells were also allowed to proliferate under influence of ConA and it was always demonstrated that CD8^+^ T cells were proliferated in greater extent when it was exposed to NLGP-TME only. NLGP-TME significantly enhances proliferation of T cells in comparison to PBS-TME (Figure 5B).

### NLGP normalized TME induces greater tumor cell cytotoxicity by effector T cells

CD8^+^ T cells were purified from NLGP-TME or PBS-TME exposed MNCs, and then subjected to cytotoxic reactions to sarcoma and lymphoma cells. PBS-TME (prepared from day 20 tumors) exposed T cells exhibited minimum cytotoxicity to sarcoma cells. However, NLGP-TME exposed CD8^+^ T cells showed greater extent of cytotoxicity to sarcoma (Figure 5C). In our previous experiments, it was observed that NLGP directs type 1 cytokine milieu by downregulating type 2 cytokines within TME. These observations prompted us to investigate whether the soluble mediators derived from NLGP-TME are sufficient to re-program the tumor associated unresponsiveness of CD8^+^ T cell functions, especially cytotoxic functions. To assess this possibility normal mouse MNCs were cultured for 120 hrs in presence of PBS-TME, with or without neutralization of different cytokines. Poor cytotoxic ability of PBS-TME exposed CD8^+^ T cells was partially recovered following neutralization of TGFβ, VEGF, IL-10 and IL-6 in the system. Use of either (VEGF+TGFβ) or (TGFβ+IL-10) combination for neutralization further enhances cytotoxic ability of PBS-TME exposed T cells (Figure 5D). Measurement of IFNγ revealed promotion of secretion of this cytokine from effector CD8^+^ T cells (Figure 5E). Interestingly, such result correlated well with cytotoxic functions of same cells. On the otherhand, neutralization of IL-12 showed a marked decrease in cytotoxic ability of PBS-TME exposed CD8^+^ T cells (Figure 5D). Overall data suggests that NLGP might maintain optimum T cell functions within TME by downregulating TGFβ and other type 2 cytokines.

### NLGP protects CD8^+^ T cells from anergy

**Figure 6:**
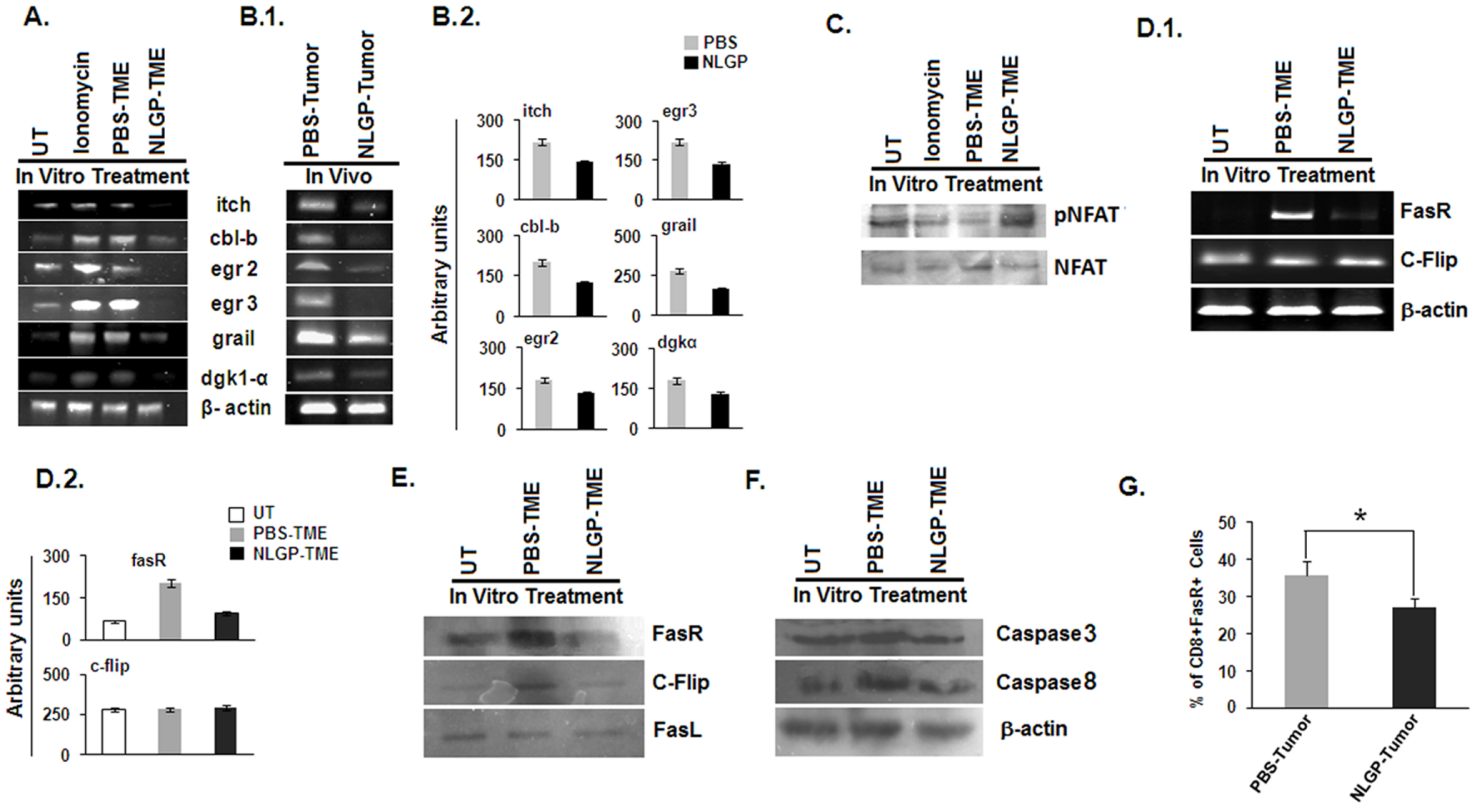
NLGP protects CD8^+^ T cells from anergy within TME. MNCs were isolated from normal mice and exposed to PBS-TME/NLGP-TME for 120 hrs and ionomycin for 48 hrs, as a positive control *in vitro*. **A.** Then CD8^+^ T cells were purified by MACS to isolate total RNA. RT-PCR analysis was performed for different anergy related genes, **B.1.** CD8^+^ T cells were directly purified from tumors of PBS and NLGP treated mice in *in vivo* condition and total RNA was isolated. Different anergy related genes were analyzed at transcriptional level by RT-PCR, **B.2.** Densitometric analysis was performed in each case. **C.** pNFAT and NFAT were analyzed at protein level, purified from CD8^+^ T cells as mentioned in A, by Western blotting **D.1.** MNCs were isolated from normal mice and exposed to PBS-TME and NLGP-TME for 120 hrs *in vitro*. Then CD8^+^ T cells were purified by MACS to isolate total RNA and protein. RT-PCR analysis was performed for Fas-R, cFlip, **D.2.** Densitometric analysis was performed in each case. **E.** and Western blot for Fas-R, cFlip, FasL, **F.** and activated Caspase3 and Caspase8. **G.** MNCs were purified from tumors of PBS and NLGP treated mice and assessed for FasR^+^CD8^+^ T cells by flow cytometry. *p<0.001.

Activated T cells may be anergized within TME. Such hyporesponsive state of T cells is activated through the T cell antigen receptor in the absence of appropriate co-stimulatory signals as well as multiple TCR signaling [30]. Upregulated expression of diacylglycerol kinases (DGKs) diminishes Ras activation, which in turn inactivates IL-2 production. Over expression of DGKs is possibly transcriptional and is accompanied by increased expression of additional negative regulators, including the transcription factors Egr (early growth response) 2 and Egr3, and the E3 ubiquitin ligases known as genes related to anergy in lymphocytes (GRAIL) and Casitas B-cell lymphoma-b (Cbl-b). As NLGP-TME induces effective CD8^+^ T cell response, next, we sought to elucidate the role of anergic process in T cell activation and its modulation by NLGP. For this purpose, we have evaluated the status of anergy related genes by RT-PCR during exposure of normal CD8^+^ T cells to PBS-TME and NLGP-TME. Results obtained from RT-PCR analysis demonstrated that purified CD8^+^ T cells, exposed to NLGP-TME, have shown negligible expression of cbl-b, egr2, egr3, itch, GRAIL and DGK1α compared to T cells exposed to PBS-TME and ionomycin as a positive control (Figure 6A). To validate obtained data, CD8^+^ T-cells were MACS purified from day 20 tumors from NLGP and PBS treated mice to obtain mRNA profile. Similar trend of results were obtained as seen in case of TME exposed T cells (Figure 6B.1 and B.2). Western blotting data showed the presence of greater amount of *p*NFAT in NLGP-TME exposed CD8^+^ T cells, in comparison to PBS-TME and ionomycin exposed control cells (Figure 6C). This data clearly provides evidence on NLGP protection within TME by preventing T cells from tumor induced anergy.

### NLGP protects CD8^+^ T cells from AICD by down regulating Fas, FasL expression

Activation induced cell death (AICD) of T cells can be an obstruction towards achieving a strong, long lived CTL response after adoptive T cell transfer in cancer immunotherapy [29]. Blockade or down regulation of CD25^+^ cells can protect T cells from AICD [31]. We have reported earlier that NLGP downregulates CD25 marker within CD4^+^ T cells [19]. Here, we observed that CD25^+^ cells are downregulated in NLGP-TME and at the same time NLGP treated T cells show greater cytotoxicity and proliferation, thus, may protect T cells from AICD. To test this hypothesis further, we have studied different key regulatory molecules in the signaling cascade important for AICD in gene as well as protein levels. CD8^+^ T cells either received no treatment or exposed to NLGP-TME expressed lower content of FasR mRNA compared to those exposed to PBS-TME, whereas, the level of mRNA content of cFLIP remains same for all three groups (Figure 6D.1 and D.2). Obtained western blot data on corresponding proteins confirm the observations on mRNAs (Figure 6E). These data supports our view that within NLGP-TME CD8^+^ T cells remain protected from AICD in significant extent. In order to ascertain the magnitude of T cell death, we have performed western blotting to measure activated level of Caspase 3 and Caspase 8 in CD8^+^ T-cells, exposed to TME either from NLGP or PBS treated mice. NLGP-TME is able to restrict activation of Caspase 3 as well as Caspase 8, which in turn gives an additional support in favour of our hypothesis (Figure 6F). Finally, we directly measured the level of AICD by measuring FasR^+^CD8^+^ T cells within tumors from PBS and NLGP treated mice. Flow cytometric evidences exhibit lower proportion of FasR^+^CD8^+^ T cells in tumors from NLGP treated mice, in comparison to control tumors (Figure 6G). All these experimental evidences suggest that NLGP can protect CD8^+^ T cells within TME from AICD, thereby, maintain their normal functionality.

### NLGP-TME educated CD8^+^ T cells migrate better to tumor site

**Figure 7:**
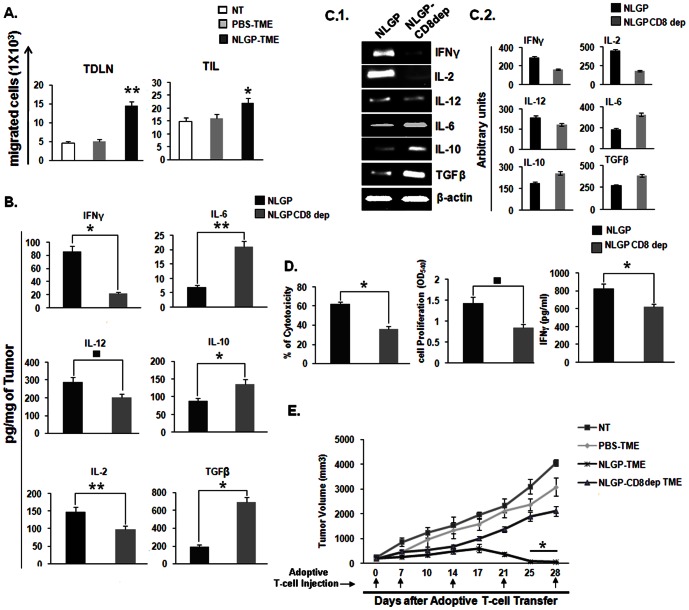
NLGP enhances T cell migration to TDLN and TIL to effectively kill tumors *in vivo*. A. MNCs were isolated from normal mice and exposed to PBS-TME and NLGP-TME for 120 hrs, along with a set of unexposed cells. Then CD8^+^ T cells were purified by MACS. Purified cells were labeled with CFSE and injected into three groups of mice having tumors of identical volume. After 24 hrs migration of CD8^+^CFSE^+^ cells were detected in TDLN and TIL, **p<0.001, *p = 0.024. **B.** Depletion of CD8 impairs NLGP mediated TME normalization. Tumor tissue lysates, representing TME from either NLGP or CD8 depleted NLGP treated mice (n = 4 in each case) were assessed for IFNγ, IL-2, IL-12, IL-6, TGFβ and IL-10 by ELISA. Cytokines were quantitated as pg/mg of tumor tissue ± SE , *p = 0.009, **p = 0.008, ^▪^ *p = *0.005, in comparison to PBS treated tumor on day 20. **C.1.** Total RNA was isolated from tumor of PBS and NLGP treated mice (n = 4 in each case) to analyze genes of IFNγ, IL-2, IL-12, IL-6, TGFβ and IL-10 by RT-PCR. **C.2.** Densitometric analysis was performed in each case. **D.** MNCs were isolated from normal mice and exposed to NLGP-TME and CD8 depleted NLGP-TME for 120 hrs. Then CD8^+^ T cells were purified by MACS and T-Cell proliferation, IFNγ release and cytotoxicity towards Sarcoma180 were measured *p = 0.01, ^▪^ *p = *0.007, **E.** MNCs were isolated from normal mice and exposed to PBS-TME, NLGP-TME and CD8 depleted NLGP-TME for 120 hrs, along with a set of unexposed cells. Then CD8^+^ T cells were purified by MACS and activated CD8^+^ T cells (1×10^7^ cells) were adoptively transferred through tail vein into four groups of tumor bearing mice (n = 6 in each group) once weekly as described in Materials and Methods (n = 3). Mean tumor volume ± SD and survivability are presented, *p<0.001.

We have provided evidences that tumor infiltrating CD8^+^ T cells are educated *in situ* for tumor cytotoxicity. Peripheral T cells need to be moved to tumor site to meet the demand. In order to check whether NLGP-TME has any additional driving efficacy, CD8^+^ T cells from healthy mouse were exposed to PBS-TME and NLGP-TME and labeled with CFSE for intravenous inoculation to tumor bearing mice. Tumors and TDLNs were harvested to check the infiltration of CD8^+^CFSE^+^ T cells. Flow cytometric data suggested that T cells exposed to NLGP-TME have greater migratory ability to tumor draining lymph nodes, thereby, to tumor compartment (Figure 7A). NLGP may regulate some chemokine signaling as discussed in earlier section within TME that drives more number of T cells to tumor. NLGP induced CXCR3 upregulation might play important role in T cell homing.

### *In vivo* CD8^+^ T cell depletion further confirms its role in NLGP mediated TME normalization

Above discussion confirms the fact that NLGP is efficient to normalize the TME and such normalized TME induces optimum T cell functions. Here, we wanted to see whether NLGP could modulate TME in CD8^+^ T cell deficient mice. For this purpose T cells are depleted in vivo using anti-CD8 antibodies one day earlier of NLGP treatment and tumors were harvested at day 20. Tumor lysates and total RNA were prepared as described before and status of regulatory cytokines/growth factors was studied at transcriptional as well as protein level. Obtained data clearly suggest that upon depletion of CD8^+^ T cells a significant distortion regarding TME normalization by NLGP was observed which clearly indicates role of CD8^+^ T cells in normalization of TME (Figure 7B–D).

### NLGP-TME educated CD8^+^ T cells participate in regression of established tumor *in vivo*

In an objective to validate the *in vitro* results, CD8^+^ T cells were either exposed to PBS-TME (Gr. 1) or NLGP-TME (Gr. 2) or none (Gr. 3) or NLGP-TME-CD8^+^ deplete (Gr. 4) (prepared from NLGP treated CD8^+^ T cell depleted mice) and injected intravenously into mice with established sarcoma (average tumor volume 256 mm^3^). Obtained results clearly showed that mice of Gr. 1 (mean TV 3077.6 mm^3^ on day 28; mean survival 45 days) and Gr. 3 have progressive tumor (mean TV 4063.5 mm^3^ on day 28; mean survival 40 days), and Gr. 4 have progressive tumor (mean TV 2109 mm^3^ on day 28; mean survival 45 days). On the other hand, all Gr. 2 mice survived till day 60, with minimum tumor load. Interestingly, all the mice with established tumor become tumor free on day 25, following adoptive transfer of NLGP-TME exposed T cells (Figure 7E).

## Discussion

Remodeling of tumor microenvironment by tumor derived factors alters the tumor-stroma architecture that favors aberrant angiogensis and the formation of an oxygen starved hostile niche. Such condition helps tumor cells to acquire more aggressive malignant potential, which can bring about more devastation by being refractile to conventional chemotherapy and radiotherapy, invigorating the angiogenic and hypoxic state and augmenting various immunosuppressive mechanisms that substantially reduce anti-tumor immunity [32]. This generalized pattern of tumors' acquisition of aggressive quality and associated immune escape is largely considered in designing novel therapeutic strategies that target the TME. Similarly, we evaluated NLGP's ability to amend TME in a mice model of sarcoma broadly based on this generalization and the data presented here demonstrate that NLGP has a striking ability to restore an antitumor microenvironment against sarcoma, which is accompanied by appreciable tumor growth restriction. It merits mentioning that NLGP mediates its effect by fine tuning of both the angiogenic and immunological factors at tumor vicinity, a finding not surprising considering the recent expansion of literature demonstrating their interdependent nature [33].

Our initial interest was to quantify various angiogenic mediators (HIF1α, VEGF, TGFβ etc) and some typical immunosuppression signatures (type 2 cytokines, Tregs, MDSCs etc) in the microenvironment of sarcoma tumors and correlate their abundance to the impairment of cytotoxic T lymphocytes, as the presence of impaired T cells in tumor vicinity has been reported as a pivotal sign of poor prognosis in many cancer types [34]. Upon comparative analysis of lysates prepared from tumors harvested on different days after sarcoma inoculation, we found that sarcoma microenvironment displayed a progressive increase of angiogenic and immunosuppressive mediators accompanied by gradual attribution of cytotoxic T cells that is favorable for tumors' growth and propagation.

To assess the rectifying power of NLGP towards this tumorigenic microenvironment, we performed a series of analysis on sarcoma tumors harvested in a similar day dependant manner after NLGP was administered therapeutically, keeping PBS treated mice as a control. From the analysis of tumor lysates from NLGP treated and control groups, an anti-tumor nature of TME by NLGP was aptly evident from the following observations. 1. A distinct transition from a type 2 cytokine milieu in PBS-TME to a type 1 environment was noted in TME from NLGP treated group. Accumulation of anti-tumor type 1 cytokines like IL-12 and IFNγ with a concomitant attrition of type 2 immunosuppressive cytokines, like, IL-10 and TGFβ in NLGP treated mice consolidates our earlier studies. 2. Reduced accumulation of several mediators of hypoxia and angiogenesis, like, HIF1α, CD31, VEGF, TGFβ, VEGFR2 in NLGP-TME compared to control was well evident. As hypoxia and angiogenesis are much acclaimed target for cancer therapy, this finding holds promise and further elaboration on this effect is one of our future goal. Another important observation is that NLGP preferentially inhibit the expression of VEGF receptor, VEGFR2 but not VEGFR1. As VEGFR2 is implicated in carcinogensis and VEGFR1 is its competitive inhibitor, this finding adds more impetus to the anti angiogenic efficacy of NLGP. All together, NLGP by inhibiting VEGF, its transcription factor HIF1α and its receptor VEGFR2 potentially restricts the formation of a hypoxic state in the tumor vicinity that may suppress atypical angiogenesis and metastasis. 3. NLGP treatment attenuated CD4^+^ CD25^+^Foxp3^+^ Tregs and Gr1^+^CD11b^+^ MDSCs compared to PBS-TME. As we have done a comprehensive study on Tregs earlier [18], this finding substantiates the earlier one and a similar study on MDSCs is envisaged based on this observation. NLGP also helps to prevent TME induced conversion of anti-tumor M1 macrophages to pro-tumor M2 type. As this is the subject of separate detail investigation this data is not included here. 4. NLGP significantly improved the expression level of chemokine receptor CXCR3 and CCR5 and their corresponding ligands that are frequently altered at tumor vicinity, thereby, stimulating proper homing of lymphocytes at tumor site. Moreover, this reinforces our earlier studies exclusively done on these chemokines [14]. We also observed a marked down-regulation of CXCR4, and its ligand SDF1α in NLGP-TME than PBS-TME.

Inhibition of the angiogenic and immunosuppressive network in the TME by NLGP culminated in the restoration of cytotoxic T cell functions. This is elaborated by studying following aspects. 1. NLGP treatment enhanced the expression of T cell activation marker CD69, two cytotoxic mediators, e. g., perforin and granzyme B, two type 1 signature cytokines, IFNγ and IL-2 and reduced IDO and CTLA4 that augment T cell impairment, compared to controls. 2. To further validate our *in vivo* results, we performed some *in vitro* assays in which splenocytes obtained from healthy mice were incubated with tumor lysates from mice treated with NLGP and PBS. T cells exposed to NLGP-TME proliferated better in presence of ConA, secreted more IFNγ and showed greater lytic ability towards sarcoma cells compared to PBS-TME as a control. Moreover, neutralization of various type 2 cytokines and growth factors (TGFβ, VEGF, IL-10 and IL-6) in different combination in PBS-TME restored the lytic ability of T cells, which indicates that NLGP induces its therapeutic effect by reducing these angiogenic and immunosuppressive mediators and NLGP-TME containing low amount of these mediators maintain normal T cell homeostasis. 3. Anergy and AICD is the terminal fates of impaired T cells where the various tumor promoting mechanisms discussed here essentially converge, accordingly, evaluated in detail. Anergy is a hyporesponsive state of T cells commonly encountered at tumor vicinity that is mediated by poor co-stimulation in absence of IL-2, a condition that upregulates various anergy related genes cbl-b, egr2, egr3, itch, GRAIL and DGK1α in T cells. Negligible expression of these anergy related genes along with higher level of phosphorylated NFAT in T cells incubated with NLGP-TME as well as those recovered from *in vivo* NLGP treated tumors compared to PBS-TME and ionomycin treated controls was a noteworthy observation. AICD causes premature turnover of T cells at tumor site and is marked by enhanced expression of FasR, caspase 3 and 8. T cells incubated with NLGP-TME showed a diminished expression of these markers and decreased expression of FasR on CD8^+^ T cells isolated from NLGP treated tumors compared to control, further confirmed the *in vitro* results. 4. Lastly, after *in vivo* administration, NLGP-TME exposed CFSE labeled T cells displayed substantial homing (accumulation of CFSE^+^ cells) at tumor draining lymph nodes and tumor sites compared to control. More accumulation of NLGP-TME exposed CD8^+^ T cells in TDLN suggests T cells are migrating in perfect route to reach in the tumor. Comparatively, low accumulation of such cells in tumors may indicate death of T cells, just after tumor killing. CD8^+^ T cells are being activated by the NLGP-TME, thus, migrated faster to TDLN. On the otherhand, T cells exposed to the PBS-TME are anergized and unable to migrate. Significant tumor killing was evident from adoptive transfer experiments, where minimum tumor was observed where T cells exposed to NLGP-TME were administrated. Accumulated evidences strongly suggest that NLGP normalized TME promotes T cells to exhibit its anti-tumor effector functions. To further confirm the participation of CD8^+^ T cells, tumor bearing mice was depleted for this particular type of cells before NLGP treatment. Tumor growth restriction by NLGP-TME educated T cells was partially disappeared in those mice depleted for CD8^+^ T cells. To explore the fact, TME of NLGP treated CD8 depleted mice were analyzed for their cytokine profile. NLGP influenced normalization of TME was significantly hampered due to depletion of CD8^+^ T cells. This observation suggests that active participation of CD8^+^ T cells is essentially required for TME normalization by NLGP. However, complete elucidation of the mechanism needs further study, especially by analyzing signaling cascades during interaction between CD8^+^ T cells, tumor cells and stromal cells under influence of NLGP. Although it is clear from our several study that NLGP directly affect different immune cells to organize observed antitumor response, still it is unexplored that how the NLGP interacts with these immune cells to cause the observed changes. Our preliminary results with flow cytometry and immunofluorescence studies using fluorescence labeled NLGP suggest NLGP might have membrane receptors on different cell types, including CD8^+^ T cells *(unpublished observation)*. Elaborate study in such direction is in progress.

A conglomeration of therapeutic monoclonal antibodies, cytokines, small molecule inhibitors and activators and dendritic cell maturation agents are often envisioned in designing effective combination therapy regimens to repair the multiple deformities in tumor *in situ* as well as in periphery of cancer patients. As we have documented in this study, NLGP's broad range of action arguably singles it out as a non toxic alternative to the potentially toxic combination therapy regimens at least for the cancer patients with lower stage of disease. Although present study is conducted on sarcoma, this is not a sarcoma specific effect. We also observed the restriction of the growth of B16 melanoma, Ehrlich's carcinoma by imparted immunomodulation of NLGP. Here, we are focused on sarcoma and its microenvironment, but similar dissection of the corresponding microenvironments is in progress in other tumor models.

The ability to normalize several parameters presupposes NLGPs ability to regulate a common denominator that orchestrates these phenomenons. Mounting evidences suggest that STAT3 is a ubiquitous regulator that negatively regulates the type 1 immune response, promotes expansion of MDSCs and Tregs and expression of angiogenic factors in the TME. Our earlier studies depict NLGP's ability to inhibit this crucial regulator [19] and we infer that inhibition of STAT3 may underpin NLGP's far reaching immunomodulatory and the inter-reliant anti-angiogenic capacities. Furthermore, NLGP may be one of the much adored STAT3 inhibitors.

The impact of the immune contexture of concerned tumors considered as immunogenic, such as melanoma or renal cell cancer, in which the success of active IL-2, IFNα or TIL immunotherapy had been documented [35], [36], as well as tumors in which there is, so far, no success of these approaches [36], which leaves open the search for alternative novel immunotherapies. Our results strongly suggest that NLGP may be one such alternative therapy. Its immunomodulatory properties coupled with the interrelated anti-angiogenic ones is enviable and we predict that the outcome of its immunomodulatory activities at tumor vicinity may be more promising in case of more immunogenic tumors. Additionally, this study sheds more light on the status of sarcoma microenvironment and may help in designing proper therapies for sarcoma in future.

To summarize, this study, besides reinforcing the powerful immunomodulatory efficacies of NLGP, shows comprehensively its diverse effects directly at the tumor site and the results are appealing for a clinical trial.

## Materials and Methods

### Reagents and antibodies

RPMI 1640 and fetal bovine serum (FBS) were purchased from Life Technologies (NY, USA). Lymphocyte separation media (LSM) was obtained from MP Biomedicals (Solon, OH, USA). CD4-FITC/Cy-chrome, Foxp3-PE and GR1-FITC mAbs were procured from BD Pharmingen (San Diego, CA, USA). CD25 (PE), CD69 (FITC), FasR (PE) were purchased from Biolegends (San Diego, California, USA). Neutralizing mAbs for VEGF, TGFβ, IL-10, IL-6, IL-12 and purified anti-mouse IL-10, IL-12, TGFβ, VEGF mAbs were obtained from e-Biosciences (San Diego, CA, USA). Fluorescence- or peroxidase-labeled secondary antibodies were also procured from e-Biosciences. Purified anti-mouse CD31 from Biolegends (San Diego, CA, USA). Purified anti-mouse Foxp3, VEGF, VEGFR1, VEGFR2, HIF1α, NFAT/*p*NFATc3 (Ser 265), cFlip, Caspase 3, Caspase 8 was procured from Santacruz Biotech (Santa Cruz, California, USA). Tri-reagent for RNA extraction was obtained from Invitrogen (CA, USA). CytoFix/CytoPerm kit, IFNγ/IL-10 estimation kits (OptEIA™), 3,3′,5,5′- tetramethylbenzidine substrate solutions, apoptosis detection kit and Stat activation sampler kit were obtained from BD Pharmingen, USA. Cytotoxicity detection kit based on lactate dehydrogenase (LDH) release was procured from Roche Diagnostics (Mannham, Germany). AEC chromogen solution was purchased from VECTOR laboratories Inc (Burlingame, CA94010). Western lightining chemiluminescence detection kit was purchased from Pierce (Rockford, IL, USA). Magnetic activated cell sorter (MACS) for CD8^+^ T cell isolation was obtained from Miltenyi Biotec Inc., CA, USA. Reverse transcription-PCR (RT-PCR) primers were procured from MWG-Biotech AG (Bangalore, India). Carboxy fluorescein diacetate succinimidyl ester (CFSE) was from Invitrogen (Eugene, OR, USA). Ionomycin, DAPI were purchased from Sigma, St. Louis, MO, USA. CD8^+^ T cell depleting antibody (Clone 2.43) was purchased from Taconic (Petersburg, NY, USA).

### Neem leaf glycoprotein (NLGP)

Extract from neem *(Azadirachta indica)* leaves was prepared by the method as described earlier [12], [13]. Mature leaves of same size and color (indicative of same age), taken from a standard source were shed-dried and pulverized. Leaf powder was soaked overnight in phosphate buffered saline (PBS), pH 7.4; supernatant was collected by centrifugation at 1500 rpm. Neem leaf preparation (NLP) was then extensively dialyzed against PBS, pH 7.4 and concentrated by Centricon Membrane Filter (Millipore Corporation, Bedford, MA, USA) with 10 kDa molecular weight cut off. Glycoprotein present in this preparation (Neem leaf glycoprotein-NLGP) was isolated and characterized by the method described [17], [25]. The purity of NLGP was checked by Size Exclusion-HPLC (SE-HPLC) in a protein PAK 300 SW column of 7.5 mm (ID)×30 cm and the protein peaks were determined by absorption at 280 nm in a UV recorder as described [17]. Toxicological profile of NLGP was also examined as reported [15].

### Animals, tumor cells and NLGP treatment

Female Swiss mice (age, 4–6 weeks, body weight, 25 g in average) were obtained from Institutional Animal Facilities, CNCI, Kolkata, India. Autoclaved dry pellet diet (Epic Laboratory Animal Feed, Kalyani, India) and water were given *ad libitum*. Maintenance and treatment of animals were given according to the guidelines established by the Institutional Animal Care and Ethics Committee. Solid tumors were developed in Swiss mice by inoculation of Sarcoma 180 (1×10^6^) cells subcutaneously on the right flank of syngenic mice and allowed to grow as solid tumor. Sarcoma 180 cells are maintained at CNCI, Kolkata, by regular intraperitoneal passage in Swiss mice. Seven days after tumor inoculation, mice having palpable tumors were injected with NLGP (25 mg/mice/injection) and PBS as control weekly for four times in total. Growth of solid tumor (in mm^3^) was monitored weekly by caliper measurement using the formula: (width^2^×length)/2. Tumors were removed from diseased mice and used in different experiments as described below.

For *in vitro* experiments Sarcoma 180 cells were maintained in RPMI 1640 with 10% FBS, penicillin (50 units/ml) and streptomycin (50 µg/ml) at 37°C with the supply of 5% CO_2_.

### Tumor microenvironment

Tumor tissues (from both NLGP and PBS treated mice) were harvested from experimental animals and weighed. The identical weight of tumor tissues from NLGP and PBS mice was minced and exposed to repetitive freeze-thaw cycles as described [37], [38]. Prepared lysates were centrifuged at 10,000 rpm for 10 mins and supernatant was collected to use as TME. TME was designated as NLGP-TME and PBS-TME. Protein concentration of the preparation was measured by using Folin-phenol reagent [39].

### Cytokine detection assay

To quantify cytokines, solid tumors harvested at different days after tumor inoculation and NLGP-TME/PBS-TME was prepared, as described above. Secretion of different cytokines (IFNγ, IL-12p40, IL-10, IL-6, TGFβ, VEGF and IL-2) within TME was assessed by ELISA and optical density was measured at 450 nm using microplate reader (BioTek Instruments Inc., Vermont, USA).

### TME educated CD8^+^ effector T cells

Spleens were isolated from normal Swiss mice to purify splenic mononuclear cells (MNCs- 1×10^6^ cells) by density gradient centrifugation on LSM and exposed to either PBS-TME or NLGP-TME (protein concentration, 10 µg) for 120 hrs on anti-CD3 coated plate at 37°C with supply of 5% CO_2_. After incubation, non-adherent fractions were collected as effector cells. CD8^+^ T cells were purified from effector cells using MACS. In brief, non-adherent RBC depleted splenic MNCs were labeled with cocktail of antibodies (except CD8^+^ antibodies), conjugated with magnetic beads and passed through a magnetic column. Flow through enriched with CD8^+^ T cells were collected as pure T cell population. Cells were washed and used in various functional T cell assays. The isolated T cell populations exceeded 95% purity as assessed by flow cytometry.

### Functional assays for T cells

TME exposed CD8^+^ T cells were then maintained in RPMI complete medium for 72 hrs. Supernatants were analyzed for extracellular release of IFNγ by ELISA using a commercially available kit. To analyse T cell proliferation after exposure to TME, T cells were assessed by MTT colorimetric assay, as described [13].

### Induction of T cell anergy

RBC depleted splenic MNCs (1×10^6^ cells) were co-incubated with either PBS-TME or NLGP-TME for 120 hrs at 37°C with supply of 5% CO_2_ [40], along with ionomycin (1 mM) exposed MNCs as positive control for studies related to T cell anergy. Following co-incubation CD8^+^ T cells were purified from the non-adherent cellular population by MACS for RT-PCR and Western blot analysis.

### Isolation of RNA and RT-PCR analysis

Total RNA was isolated from single-cell suspension or solid tumors (for tumor single-cell suspensions were obtained by enzymatic digestion) and prepared using the Tri-reagent. The cDNA synthesis was carried out using RevertAid™ First Strand cDNA Synthesis Kit (Fermentas, K1622) following the manufacturer's protocol and PCR were carried out using gene-specific primers. The oligonucleotide primers used are listed in Table 1, Table 2, Table 3. PCR products were identified by image analysis software for gel documentation (Gel Doc™ XR+ system, BioRad) following electrophoresis on 2% agarose gels stained with ethidium bromide.

**Table 1:** Primer sequences of genes related to chemokine receptor and ligands.

Name	Primer sequences (5′-3′)	Product size
CXCR3-forward	GCTGCTGTCCAGTGGGTTTT	67 bp.
CXCR3-reverse	AGTTGATGTTGAACAAGGCGC	
CXCL9-forward	TGGGCATCATCTTCCTGGAG	204 bp.
CXCL9- reverse	CCGGATCTAGGCAGGTTTGA	
CXCL10-forward	CCAAGTGCTGCCGTCATTTT	177 bp.
CXCL10- reverse	CTCAACACGTGGGCAGGATA	
CCR5-forward	ACTGCTGCCTAAACCCTGTCA	78 bp.
CCR5-reverse	GTTTTCGGAAGAACACTGAGAGATAA	
CCL3-forward	CCTCTGTCACCTGCTCAACA	163 bp.
CCL3-reverse	GATGAATTGGCGTGGAATCT	
CCL4-forward	GCTGTGGTATTCCTGACCAAA	196 bp.
CCL4- reverse	AAATCTGAACGTGAGGAGCAA	
CCL5-forward	CCCTCACCATCATCCTCACT	186 bp.
CCL5- reverse	TCCTTCGAGTGACAAACACG	
CCL8-forward	ACGCTAGCCTTCACTCCAA	231 bp.
CCL8- reverse	TCTGGAAAACCACAGCTTCC	
CXCR4 -forward	TCAGTGGCTGACCTCCTCTT	203 bp
CXCR4 -reverse	CTTGGCCTCTGACTGTTGGT	
CXCL12-forward	CTGCATCAGTGACGGTAAACC	142 bp
CXCL12-reverse	CAGCCGTGCAACAATCTGAAG	
β-Actin-forward	CAACCGTGAAAAGATGACCC	228 bp.
β-Actin-reverse	ATGAGGTAGTCTGTCAGGTC	

**Table 2:** Primer sequences of various genes of cytokine, growth factors etc.

Name	Primer sequences (5′-3′)	Product size
IFNγ-forward	ACTGGCAAAAGGATGGTGAC	237 bp
IFNγ-reverse	TGAGCTCATTGAATGCTTGG	
IL-2- forward	GCAGGCCACAGAATTGAAAG	207 bp
IL-2- reverse	TCCACCACAGTTGCTGACTC	
IL-12- forward	CCTGCATCTAGAGGCTGTCC	243 bp
IL-12- reverse	CATCTTCTTCAGGCGTGTCA	
IL-6- forward	TTCCATCCAGTTGCCTTCTT	199 bp
IL-6- reverse	CAGAATTGCCATTGCACAAC	
IL-10- forward	CCAAGCCTTATCGGAAATGA	162 bp
IL-10- reverse	TTTTCACAGGGGAGAAAATCG	
TGFβ-forward	TGCGCTTGCAGAGATTAAAA	197 bp
TGFβ-reverse	GCTGAATCGAAAGCCCTGTA	
VEGF-forward	GGACCCTGGCTTTACTGCTG	201 bp
VEGF-reverse	CACAGGACGGCTTGAAGATG	
HIF1α-forward	TCAAGTCAGCAACGTGGAAG	198 bp
HIF1α-reverse	TATCGAGGCTGTGTCGACTG	
VEGFR2-forward	ACAGACAGTGGGATGGTCC	271 bp
VEGFR2-reverse	AAACAGGAGGTGAGCGCAG	
VEGFR1-forward	CCAACTACCTCAAGAGCAAAC	315 bp
VEGFR1-reverse	CCAGGTCCCGATGAATGCAC	
IDO1-forward	TGAAAAGCTGCCCACACTGA	300 bp
IDO1-reverse	CAGTCCCCACCAGGAAATGA	
CTLA4 -forward	TCAGTGGTGTTGGCTAGCAG	228 bp
CTLA4-reverse	CAGTCCTTGGATGGTGAGGT	
Perforin-forward	GATGTGAACCCTAGGCCAGA	161 bp.
Perforin-reverse	GGTTTTTGTACCAGGCGAAA	
Granzyme B-forward	TCGACCCTACATGGCCTTAC	198 bp.
Granzyme B-reverse	TGGGGAATGCATTTTACCAT	

**Table 3:** Primer sequences of apoptosis, anergy and AICD related genes.

Name	Primer sequences (5′-3′)	Product size
Itch-forward	CATGTGGTTTTGGCAGTTTG	216
Itch-reverse	TTGTAAGGTGGGAGGTCCAG	
Cbl-b-forward	GAGCCTCGCAGGACTATGAC	241
Cbl-b-reverse	CTGGCCACTTCCACGTTATT	
Egr2-forward	CAGGAGTGACGAAAGGAAGC	202
Egr2-reverse	ATCTCACGGTGTCCTGGTTC	
Egr3-forward	ATCCCTTCTTTTCCCTTCCA	199
Egr3-reverse	CAAGGAGAAAGTGCCTCCAG	
Grail-forward	AGAGAGAGGGGCTTCTGGAG	173
Grail-reverse	CGATGACCATTGTGACTTGG	
Dgk1α-forward	AAGGCATTGCGGTATTGAAC	160
Dgk1α -reverse	TCTGGCACACAGGTCTTCAG	
FasR-forward	CAGACATGCTGTGGATCTGG	181
FasR-reverse	CATGGTTGACAGCAAAATGG	
c-Flip-forward	TGGGCATGACTACTGTGGAA	220
c-Flip -reverse	AGGACATGAGTTCCGTGAGG	

### Western blot analysis

For tumor lysate preparation, pieces of solid tumors were frozen into liquids nitrogen and thawed in 37°C water bath for 2–3 cycle and smashed by a motor pestle. It was further dissolved in RIPA buffer incubated for 30 mins at 4°C. After that it was centrifuged at 12, 000 rpm 4°C and supernatant was taken as tumor-lysate. The tumor lysate or cellular lysate (protein concentration, 50 µg) were separated on 6–20% SDS-polyacrylamide gel and transferred onto a PVDF membrane (Millipore, USA) using the BioRad Gel Transfer system. The membrane was first blocked with the 5% BSA for 2 hr at room temperature. This was followed by incubation overnight at 4°C with the primary antibody, then, for 2 hr at room temperature with peroxidase-conjugated secondary antibody. Immunoreactive proteins were detected by addition of the HRP color development reagent according to manufacturer's protocol. The membrane was immersed into the solution for 1 min, wrapped with a Saran wrap exposed to X-ray film and developed.

### Immunohistochemical analysis

Tumor sections were mounted on poly-L-lysine coated slides and allowed to dry for 1 hr at 37°C, followed by 1 hr incubation in an incubator at 60°C. After deparaffinization and rehydration, tissue sections were treated for antigen retrieval using 0.01 (M) citrate buffer, pH 6, at 80°C for 45 mins. After washing with PBS, tissue sections were covered for 30 min with 0.3% H_2_O_2_ to block endogenous peroxidase, followed by an additional washing with the TBS-Tween-20 buffer. Slides were then placed in a humid chamber and incubated for 30 min with the blocking solution (5% BSA) followed by primary mouse antibody (anti-VEGF, VEGFR1, VEGFR2, TGFβ, HIF1α). After three rinses in PBS, slides were incubated with the HRP conjugated secondary antibody for 30 min. Tissue staining was visualized with an AEC chromogen solution. Slides were counterstained with Mayer's hematoxylin, dehydrated and mounted. Negative controls were performed by using a mouse IgG. To validate each staining, positive and negative controls were kept.

### Immunofluorescence analysis

For immunofluoroscence analysis tumor tissue samples were prepared and sectioned by the method described [41]. All washing steps were performed using 0.5% BSA in PBS while blocking steps were carried out using 2% BSA in PBS. For detection of the presence of CD31, sections were incubated with rat anti-mouse CD31 followed by FITC conjugated anti-rat antibody. All sections were counterstained with DAPI and then mounted. Images were acquired using Leica DM 1000, Fluorescent Microscope (Leica, BM 4000B, Germany).

#### Flow cytometric analysis for cellular biomarkers

Flow cytometric analysis for surface phenotypic markers on immune cells within TME (i.e., activated T cells, Tregs, MDSCs etc) was performed by preparing single cell suspension from solid tumors, then labeling with different anti-mouse fluorescence labeled antibodies (CD11b, Gr1, CD8, CD4, CD69, CD25 and Foxp3) for 30 min as per manufacturer's recommendation. After labeling, cells were washed in FACS buffer (1% FBS in PBS), fixed in 1% paraformaldehyde in PBS and cytometry was performed on a FACS Caliber flow cytometer using Cell Quest software (Becton Dickinson, Mountainview, CA). Suitable negative isotype controls were used to rule out the background fluorescence. Intra-cellular staining of Foxp3 was performed by controlled permealization of cellular membrane using Cytofix/Cytoperm™ Kit. The data was generated by cytofluorometric analyses of 10,000 events. Percentage of each positive population and mean fluorescence intensity (MFI) were determined by using quadrant statistics.

### CD8^+^ T cell migration by CFSE labeling

TME educated CD8^+^ T cells were prepared as stated before and cells were labeled with CFSE-DA (6 µM). Sarcoma tumor bearing mice (tumor volume approximately 108 mm^3^) was then adoptively transferred intravenously (through tail vein) with CFSE-DA labeled CD8^+^ T cells (1×10^7^ cells), educated with either PBS-TME or NLGP-TME. CFSE-DA labeled CD8^+^ T cells only was also kept as control. Mice were sacrificed following 24 hours of T cell transfer and single cells were isolated from TDLN and tumors. Isolated cells were labeled with PE conjugated CD8^+^ antibody and CD8^+^CFSE^+^ cells were identified by flow cytometric analysis.

### *In vitro* cytotoxicity with TME educated CD8^+^ T cells

Cytotoxicity of CD8^+^ T cells (primed with TME) against mouse sarcoma cells was tested by LDH release assay using a cytotoxicity detection kit. In brief, 1×10^4^ tumor cells were plated as target in 96-well cell culture plates. TME exposed CD8^+^ T cells were added in triplicate as effector cells in each well and incubated overnight. Cell-free supernatants were used to measure the level of released LDH using the formula: 
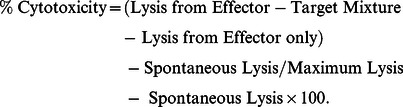



### *In vivo* depletion of T cells

*In vivo* depletion of CD8^+^ T cells sarcoma bearing mice was done by intravenous injection of CD8^+^ T cell depleting antibody (Clone 2.43) once weekly just one day before of NLGP treatment. Establishment of CD8 depletion was confirmed by flow cytometric analysis using anti- CD8-PE antibody.

### Therapy with TME educated CD8^+^ T cells

TME educated CD8^+^ T cells (1×10^7^ cells/mice) were then adoptively transferred to sarcoma bearing mice (tumor volume approximately 256 mm^3^) through tail vein. CD8^+^ T cells only were inoculated in a separate group of mice as control. Cellular therapy was given once in a week, four times in total. Tumor volume was measured by the formula given earlier and mice survival was checked in regular interval.

### Statistical analysis

All results represent the average of five separate *in vitro* experiments and three *in vivo* experiments. The number of experiments is mentioned in the result section and legends to Figures. In each experiment, a value represents the mean of three individual observations and is presented as mean ± standard deviation (SD) or standard error (SE). Statistical significance was established by unpaired t-test using INSTAT 3 Software (GraphPad Software, Inc.), with differences between groups attaining a p value<0.05 considered as significant.

## References

[1] Lorusso G, Rüegg C (2008) The tumor microenvironment and its contribution to tumor evolution toward metastasis. Histochem Cell Biol 130: 1091–1103. 18987874 10.1007/s00418-008-0530-8

[2] Prager GW, Poettler M, Unseld M, Zielinski CC (2012) Angiogenesis in cancer: Anti-VEGF escapes mechanisms. Transl Lung Cancer Res 1: 14–25. 25806151 10.3978/j.issn.2218-6751.2011.11.02 PMC4367591

[3] Witz IP (2009) The tumor–microenvironment interactions: The making of a paradigm. Cancer Microenviron 2: 9–17. 10.1007/s12307-009-0025-8 PMC2756342 19701697

[4] Egeblad M, Nakasone ES, Werb Z (2010) Tumors as organs: complex tissues that interface with the entire organism. Dev Cell 18: 884–901. 20627072 10.1016/j.devcel.2010.05.012 PMC2905377

[5] Rehman J, Li J, Orscell C, March K (2003) Peripheral blood endothelial cells are derived from monocyte/macrophages and secrete angiogenic growth factors. Circulation 107: 1164–1169. 12615796 10.1161/01.cir.0000058702.69484.a0

[6] Bhowmick NA, Neilson EG, Moses HL (2004) Stromal fibroblasts in cancer initiation and progression. Nature 432: 332–337. 15549095 10.1038/nature03096 PMC3050735

[7] Whiteside TL (2006) Immune suppression in cancer: Effects on immune cells, mechanisms and future therapeutic intervention. Semin Cancer Biol 16: 3–15. 16153857 10.1016/j.semcancer.2005.07.008

[8] Mbeunkui F, Johann DJ Jr (2009) Cancer and the tumor microenvironment: a review of an essential relationship. Cancer Chemother Pharmacol 63: 571–582. 19083000 10.1007/s00280-008-0881-9 PMC2858592

[9] Biswas K, Chattopadhyay I, Banerjee RK, Bandopadhyay U (2002) Biological activities and medicinal properties of neem (Azadirachta indica). Curr Sci 82: 1336–1345.

[10] Paul R, Prasad M, Sah NK (2011) Anticancer biology of *Azadirachta indica* L (neem) A mini review. Cancer Biol Ther 12: 467–476 21743298 10.4161/cbt.12.6.16850

[11] Fujiwara T, Takeda T, Ogihara Y, Shimizu M, Nomura T, et al (1982) Studies on the structure of polysaccharides from the bark of Melia azadirachta. Chem Pharm Bull 30: 4025–4030. 7165877 10.1248/cpb.30.4025

[12] Baral R, Chattopadhyay U (2004) Neem (Azadirachta indica) leaf mediated immune activation causes prophylactic growth inhibition of murine Ehrlich carcinoma and B16 melanoma. Int Immunopharmacol 4: 355–366. 15037213 10.1016/j.intimp.2003.09.006

[13] Baral R, Mandal I, Chattopadhyay U (2005) Immunostimulatory neem leaf preparation acts as an adjuvant to enhance the efficacy of poorly immunogenic B16 melanoma surface antigen vaccine. Int Immunopharmacol 5: 1343–1352. 15914339 10.1016/j.intimp.2005.03.008

[14] Chakraborty K, Bose A, Pal S, Sarkar K, Goswami S, et al (2008) Neem leaf glycoprotein restores the impaired chemotactic activity of peripheral blood mononuclear cells from head and neck squamous cell carcinoma patients by maintaining CXCR3/CXCL10 balance. Int Immunopharmacol 8: 330–340. 18182249 10.1016/j.intimp.2007.10.015

[15] Mallick A, Ghosh S, Banerjee S, Majumder S, Das A, et al (2013) Neem leaf glycoprotein is nontoxic to physiological functions of Swiss mice and Sprague Dawley rats: histological, biochemical and immunological perspectives. Int Immunopharmacol 15: 73–83. 23178577 10.1016/j.intimp.2012.11.006

[16] Bose A, Chakraborty K, Sarkar K, Goswami S, Chakraborty T, et al (2009) Neem leaf glycoprotein induces perforin mediated tumor cell killing by T and NK cells through differential regulation of IFNγ signaling. J Immunother 32: 42–53. 19307993 10.1097/CJI.0b013e31818e997d

[17] Goswami S, Bose A, Sarkar K, Roy S, Chakraborty T, et al (2010) Neem leaf glycoprotein matures myeloid derived dendritic cells and optimizes anti-tumor T cell functions. Vaccine 28: 1241–1252. 19969119 10.1016/j.vaccine.2009.11.018

[18] Haque E, Baral R (2006) Neem (*Azadirachta indica*) leaf preparation induces prophylactic growth inhibition of murine Ehrlich carcinoma in Swiss and C57BL/6 by activation of NK cells and NK-T cells. Immunobiology 211: 721–731. 17015147 10.1016/j.imbio.2006.02.005

[19] Chakraborty T, Bose A, Barik S, Goswami KK, Banerjee S, et al (2011) Neem leaf glycoprotein inhibits CD4+CD25+Foxp3+ Tregs to restrict murine tumor growth. Immunotherapy 3: 949–969. 21843083 10.2217/imt.11.81

[20] Bose A, Chakraborty K, Sarkar K, Goswami S, Haque E, et al (2009) Neem leaf glycoprotein directs T-bet associated type 1 immune commitment. Human Immunol 70: 6–15. 18983881 10.1016/j.humimm.2008.09.004

[21] Sarkar K, Bose A, Haque E, Chakraborty K, Chakraborty T, et al (2009) Induction of type 1 cytokines during neem leaf glycoprotein assisted carcinoembryonic antigen vaccination is associated with nitric oxide production. Int Immunopharmacol 9: 753–760. 19285575 10.1016/j.intimp.2009.02.016

[22] Chakraborty K, Bose A, Chakraborty T, Sarkar K, Goswami S, et al (2010) Restoration of dysregulated CC chemokine signaling for monocyte/macrophage chemotaxis in head and neck squamous cell carcinoma patients by neem leaf glycoprotein maximizes tumor cell cytotoxicity. Cell Mol Immunol 7: 396–408. 20622890 10.1038/cmi.2010.29 PMC4002678

[23] Sarkar K, Bose A, Chakraborty K, Haque E, Ghosh D, et al (2008) Neem leaf glycoprotein helps to generate carcinoembryonic antigen specific anti-tumor immune responses utilizing macrophage-mediated antigen presentation. Vaccine 26: 4352–4362. 18590789 10.1016/j.vaccine.2008.06.048

[24] Sarkar K, Goswami S, Roy S, Mallick A, Chakraborty K, et al (2010) Neem leaf glycoprotein enhances carcinoembryonic antigen presentation of dendritic cells to T and B cells for induction of antitumor immunity by allowing generation of immune effector/memory response. Int Immunopharmacol 10: 865–887. 20472099 10.1016/j.intimp.2010.04.024

[25] Mallick A, Barik S, Goswami KK, Banerjee S, Ghosh S, et al (2013) Neem leaf glycoprotein activates CD8+ T cells to promote therapeutic anti-tumor immunity inhibiting the growth of mouse sarcoma. PLoS ONE 8: e47434. 23326300 10.1371/journal.pone.0047434 PMC3543399

[26] Zamarron BF, Chen W (2011) Dual Roles of Immune Cells and Their Factors in Cancer Development and Progression. Int J Biol Sci 7: 651–658. 21647333 10.7150/ijbs.7.651 PMC3107473

[27] Hinrichs C, Spolski R, Paulos CM, Gattinoni L, Kerstann KW, et al (2008) IL-2 and IL-21 confer opposing differentiation programs to CD8^+^ T cells for adoptive immunotherapy. Blood 111: 5326–5333. 18276844 10.1182/blood-2007-09-113050 PMC2396726

[28] Ebert LM, Schaerli P, Moser B (2005) Chemokine-mediated control of T cell traffic in lymphoid and peripheral tissues. Mol Immunol 42: 799–809. 15829268 10.1016/j.molimm.2004.06.040

[29] Lu B, Finn OJ (2008) T-cell death and cancer immune tolerance. Cell Death Differ 15: 70–79. 18007660 10.1038/sj.cdd.4402274

[30] Zheng Y, Zha Y, Gajewski TF (2008) Molecular regulation of T-cell anergy. EMBO ep 9: 50–55. 10.1038/sj.embor.7401138 PMC2246614 18174897

[31] Wood KJ, Sakaguchi S (2003) Regulatory T cells in transplantation tolerance. Nat Rev Immunol 3: 199–210. 12658268 10.1038/nri1027

[32] Murdoch C, Muthana M, Coffelt SB, Lewis CE (2008) The role of myeloid cells in the promotion of tumour angiogenesis. Nat Rev Cancer 8: 618–663. 18633355 10.1038/nrc2444

[33] Kerbel RS (1991) Inhibition of tumor angiogenesis as a strategy to circumvent acquired resistance to anti-cancer therapeutic agents. Bioessays 13: 31–36. 1722975 10.1002/bies.950130106

[34] Xu L, Xu W, Qiu S, Xiong S (2010) Enrichment of CCR6(+)Foxp3(+) regulatory T cells in the tumour mass correlates with impaired CD8(+) T cell function and poor prognosis of breast cancer. Clin Immunol 135: 466–475. 20181533 10.1016/j.clim.2010.01.014

[35] Stadler WM, Kuzel T, Dumas M, Vogelzang NJ (1998) Multicenter phase II trial of interleukin-2, interferon-alpha, and 13-cis-retinoic acid in patients with metastatic renal-cell carcinoma. J Clin Oncol 16: 1820–1825. 9586896 10.1200/JCO.1998.16.5.1820

[36] Rosenberg SA, Spiess P, Lafreniere R (1986) A new approach to the adoptive immunotherapy of cancer with tumor-infiltrating lymphocytes. Science 233: 1318–1321. 3489291 10.1126/science.3489291

[37] Yamanaka R, Abe T, Yajima N, Tsuchiya N, Homma J, et al (2003) Vaccination of recurrent glioma patients with tumour lysate-pulsed dendritic cells elicits immune responses: results of a clinical phase I/II trial. Br J Cancer 89: 1172–1179. 14520441 10.1038/sj.bjc.6601268 PMC2394324

[38] Yu J, Tian R, Xiu B, Yan J, Jia R, et al (2009) Antitumor activity of T cells generated from lymph nodes draining the SEA-expressing murine B16 melanoma and secondarily activated with dendritic cells. Int J Biol Sci 5: 135–146. 19173035 10.7150/ijbs.5.135 PMC2631223

[39] Bailey JL (1967) Miscellaneous analytical methods. In: Bailey JL, editor. Techniques in Protein Chemistry. New York: Elsevier Science Publishing 340–346.

[40] Hayashi RJ, Loh DY, Kanagawa O, Wang F (1998) Differences between responses of naive and activated T cells to anergy induction. J Immunol 160: 33–38. 9551953

[41] Komita H, Zhao X, Taylor JL, Sparvero LJ, Amoscato AA, et al (2008) CD8+ T-cell responses against hemoglobin-b prevent solid tumor growth. Cancer Res 68: 8076–8084. 18829566 10.1158/0008-5472.CAN-08-0387 PMC2597529

